# Unexpected twist: large marginal branch occlusion of the left circumflex artery unveiled by immediate and pivotal cardiac magnetic resonance imaging in a 19-year-old with suspected myocarditis—a case report

**DOI:** 10.1093/ehjcr/ytag015

**Published:** 2026-01-22

**Authors:** Sebastian N Nagel, Wladimir N Tschishow, Ziyad Alomari, Carsten W Israel, Günther Wittenberg

**Affiliations:** Department of Radiology, Evangelisches Klinikum Bethel, University Hospital OWL, Bielefeld University, Burgsteig 13, Bielefeld 33617, Germany; Department of Internal Medicine, Cardiology, Nephrology and Diabetology, Evangelisches Klinikum Bethel, University Hospital OWL, Burgsteig 13, Bielefeld 33617, Germany; Department of Internal Medicine, Cardiology, Nephrology and Diabetology, Evangelisches Klinikum Bethel, University Hospital OWL, Burgsteig 13, Bielefeld 33617, Germany; Department of Internal Medicine, Cardiology, Nephrology and Diabetology, Evangelisches Klinikum Bethel, University Hospital OWL, Burgsteig 13, Bielefeld 33617, Germany; Department of Radiology, Evangelisches Klinikum Bethel, University Hospital OWL, Bielefeld University, Burgsteig 13, Bielefeld 33617, Germany

**Keywords:** Endocarditis, Coronary artery occlusion, Cardiac MRI, Myocarditis, Septic embolism, Streptococcus mitis, Case report

## Abstract

**Background:**

Myocarditis and myocardial infarction in young patients can present with overlapping symptoms, posing a diagnostic challenge. Advanced cardiac imaging, particularly cardiac magnetic resonance imaging (MRI), plays a pivotal role in distinguishing between these entities.

**Case summary:**

A 19-year-old male presented with a 5-week history of fever, general weakness, and new atypical chest pain, raising the suspicion of myocarditis. Immediate cardiac MRI revealed signs of lateral wall ischaemia. Coronary angiography confirmed an occlusion of a large marginal branch of the left circumflex artery. Pathological analysis of the retrieved thrombus indicated a septic embolism, and further investigations confirmed endocarditis due to *Streptococcus mitis*.

**Discussion:**

This case highlights the essential role of early cardiac MRI in guiding clinical decision-making, particularly in young patients with non-specific symptoms. Although rare, endocarditis-related septic embolism must be considered in the differential diagnosis of acute myocardial infarction in this population.

Learning points

- Cardiac MRI can play a decisive role in differentiating ischaemic from inflammatory myocardial injury, even when no ischaemia-specific protocol is applied.
- Septic coronary embolism should be considered in patients with endocarditis, as it is a rare but potentially life-threatening cause of myocardial infarction.

## Introduction

Myocarditis and myocardial infarction in young patients often present with overlapping clinical symptoms, posing a diagnostic challenge.^1,2^ Advanced imaging, such as cardiac magnetic resonance imaging (MRI), plays a pivotal role in distinguishing these conditions, ensuring timely and appropriate treatment.^3^ Here, we report an unusual case of a large marginal branch (MB) occlusion of left circumflex artery (LCX) caused by a septic embolism in a young male, initially suspected of having myocarditis, where immediate cardiac MRI was instrumental in guiding further management.

## Summary figure

**Figure:**
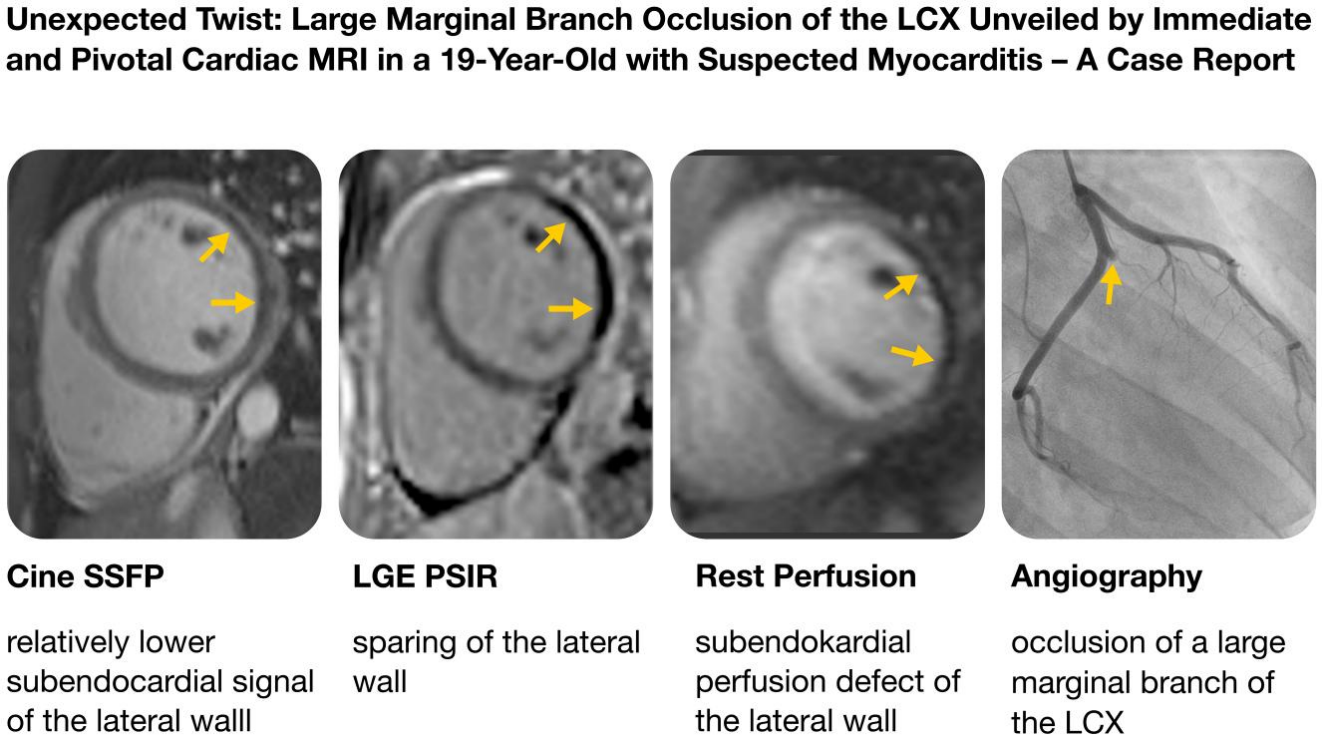


## Case presentation

### Patient history and presentation

A 19-year-old male presented to the emergency department with a 5-week history of fever (37.6–40°C), a feeling of general weakness, and new-onset of atypical chest pain. The patient reported a heart valve defect but could not provide further details; apart from borreliosis at the age of five, the medical history was otherwise uneventful.

### Initial diagnostic workup and findings

**Figure 1:**
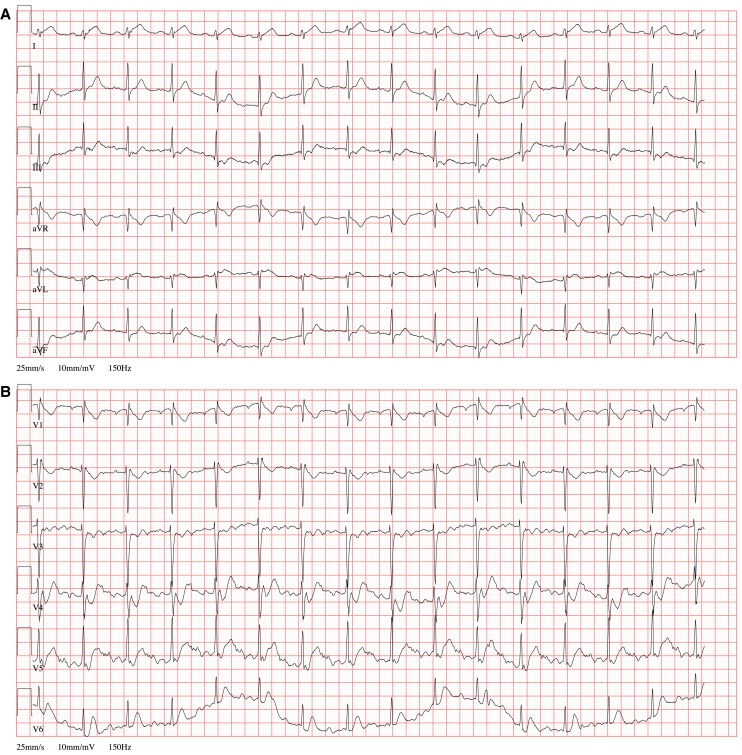
(*A* and *B*) Electrocardiogram (25 mm/s, 10 mm/mV) on admission showing subtle changes, including mild ST-segment elevation (∼1 mm) in leads I and aVL, with reciprocal ST-segment depression in the inferior leads. While not diagnostic, these alterations may be consistent with early or subtle ischaemic changes.

**Figure 2:**
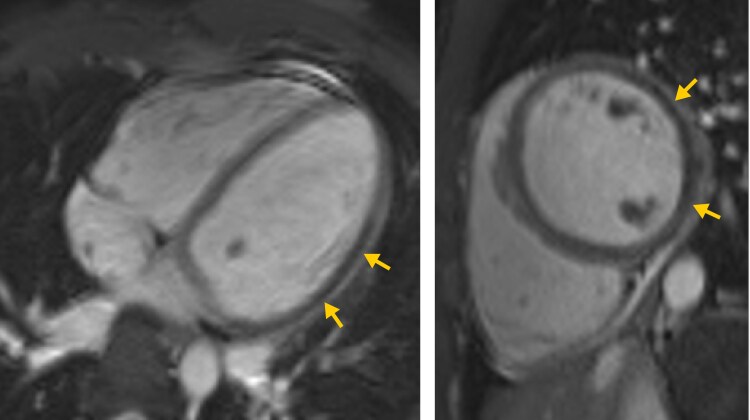
Cine-SSFP images: (*A*) four-chamber and (*B*) short-axis view showing relatively lower signal in the lateral wall (yellow arrows).

**Figure 3:**
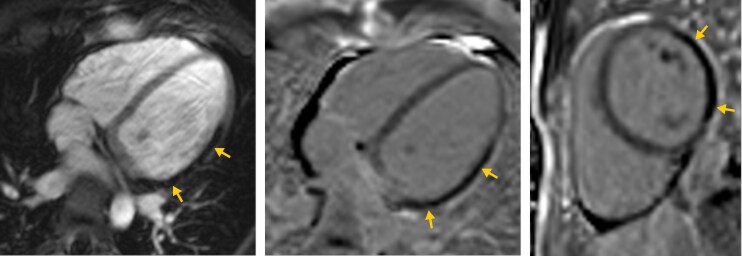
Gadolinium enhancement images: (*A*) four chamber early gadolinium enhancement magnitude image as well as late gadolinium enhancement phase sensitive inversion recovery image in the (*B*) four-chamber and (*C*) short-axis view showing no contrast uptake in the lateral wall (yellow arrows).

**Figure 4:**
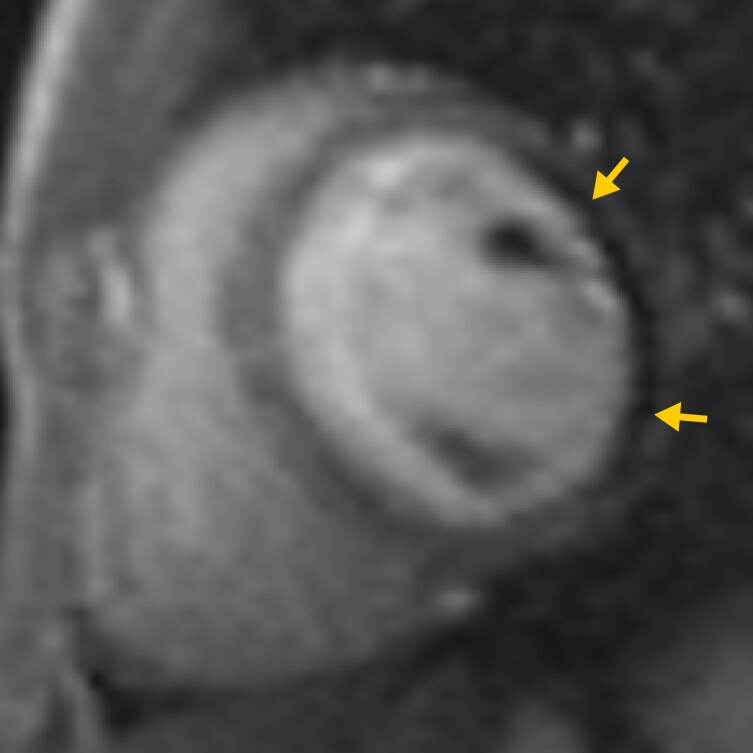
Rest perfusion imaging confirming perfusion defect in the lateral wall (yellow arrows).

Vital signs on admission showed fever of 38.6°C, and C-reactive protein was elevated to 48.7 mg/L. Electrocardiogram on admission showed subtle but noteworthy changes, including approximately 1 mm ST-segment elevation in leads I and aVL with reciprocal 1 mm ST-segment depression in the inferior leads (*Figure 1*); although the baseline was slightly unstable and the findings were not diagnostic for acute myocardial infarction, they may be compatible with early or subtle ischaemic alterations. Troponin-T (high sensitive) rose from 64 to 89 pg/mL within 1 h. Transthoracic echocardiography in the emergency department revealed a mitral valve insufficiency with a prominent prolapse of the posterior mitral leaflet (P2 segment), where the latter could not be conclusively interpreted at that time (see Supplementary material online, *Video S1*). In light of these observations, a cardiac MRI was performed to investigate the possibility of myocarditis (Avanto 1.5 Tesla MRI, Siemens Healthineers, Erlangen, Germany using a total of 14 mL of Gadovist, Bayer Vital, Leverkusen, Germany). Diagnostic sequences included Cine-SSFP, TIRM images, as well as early and late gadolinium enhancement images (EGE, LGE). Notable findings comprised signal alterations in the lateral wall on Cine-SSFP sequences (*Figure 2*), where EGE and LGE images showed complete absence of signal in the lateral wall (*Figure 3*). A spontaneously added rest perfusion sequence confirmed this as a perfusion deficit (*Figure 4*). These findings strongly suggested a coronary occlusion. Of note, the vegetation that was later confirmed on the mitral valve is visible on the two-chamber long-axis view (see Supplementary material online, *Video S2*).

### Clinical course and interventions

**Figure 5:**
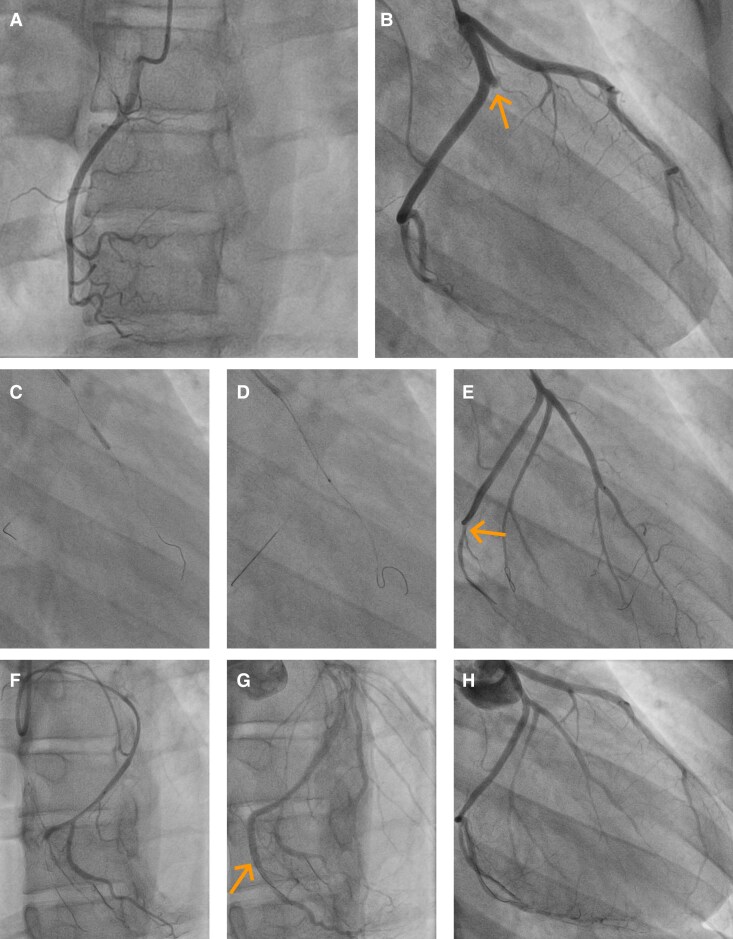
Panels (*A*)–(*H*) are shown in chronological order. (*A*) Coronary angiography of the right coronary artery showing no relevant abnormalities. (*B*) Occlusion of a large marginal branch of the left circumflex artery (arrow). (*C–E*) Fragmentation and aspiration of the thrombus; in (*E*), antegrade perfusion is restored, but a new distal filling defect in the left circumflex artery is visible (arrow). (*F–H*) Fragmentation and aspiration of the left circumflex artery thrombus are performed; as perfusion could not be fully restored, the affected segment is finally treated with stent implantation (arrow). Note the fractured tip of one of one of the guidewires in (*F*).

Based on the MRI findings, the patient was referred for immediate coronary angiography, which confirmed occlusion of a large MB of the LCX (*Figure 5B*). The thrombus was first fragmented and then retrieved using an aspiration catheter (Eliminate™ Aspiration Catheter, Terumo, Shibuya, Japan), what lead to restored perfusion. During retrieval of the thrombus, fragments embolized to the distal LCX, where complete perfusion could not be restored by fragmentation and aspiration and the segment was stented with a 2.75 × 16 mm drug-eluting stent (DES) (Promus Premier Select, Boston Scientific, Marlborough, MA, USA). Notably, during the procedure, the tip of one of the guidewires fractured in a marginal branch, and the affected segment was subsequently secured with an additional 2.5 × 8 mm DES (Promus Premier Select, Boston Scientific, Marlborough, MA, USA).

As the aspirated thrombus appeared tissue-like rather than resembling a typical blood clot, pathological analysis was initiated and confirmed a septic embolism as the underlying aetiology. A transoesophageal echocardiography was performed, which again showed mitral valve insufficiency and led to re-interpretation of the initially unclear prolapse as a probable vegetation. An additional MRI of the neurocranium showed small disseminated infarcts consistent with thromboembolic origin. Given these findings, the patient was transferred for mitral valve reconstruction surgery.

The final diagnosis of endocarditis was established by fever, the history of an unknown heart valve defect, the second echocardiography (mitral insufficiency + vegetation), microbiological analysis of the aspirated thrombus, and perioperative histological confirmation from surgical specimens. The microbiological workup revealed *Streptococcus mitis* as the causative organism.

The patient was contacted approximately 3 months later to obtain consent for this publication. He reported good recovery and well-being.

## Discussion

This case underscores the diagnostic challenges in young patients presenting with fever and atypical chest pain, where myocarditis is frequently considered the primary differential diagnosis.^1,2^ In this scenario, immediate cardiac MRI proved pivotal in identifying myocardial ischaemia and redirecting the clinical pathway towards coronary angiography. Despite being considered a rare complication of infective endocarditis, and although a comprehensive review of the literature is beyond the scope of this report, several cases of coronary artery embolism resulting in myocardial infarction have been described.^4–13^ Notably, post-mortem studies have demonstrated myocardial infarction or coronary embolism in up to 40% of patients with endocarditis, suggesting that this complication may be under-recognized in clinical practice.^14^

## Conclusion

This case highlights the essential role of cardiac MRI in differentiating ischaemic from inflammatory myocardial conditions in young patients. Prompt imaging can significantly impact clinical decision-making and patient outcome.

## Lead author biography


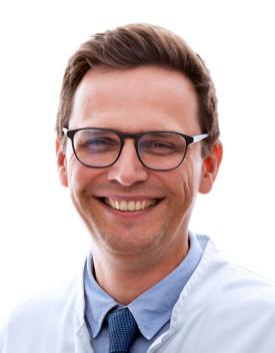


Sebastian Nagel is a senior radiologist at the Evangelisches Klinikum Bethel in Bielefeld, Germany. He completed his medical degree and doctorate at Charité – Universitätsmedizin Berlin, where he also obtained board certification in radiology and completed his habilitation. During his time at Charité, he focused primarily on cardiac imaging. His main interests include the clinical application of advanced imaging techniques. He is also a fellow of the European Society of Radiology (EDiR).

## Supplementary Material

ytag015_Supplementary_Data

## Data Availability

The data underlying this article cannot be made publicly available, as full consent for unrestricted access was not obtained from the patient. However, data may be provided upon reasonable request to the corresponding author.

## Supplementary material

**Consent:** The authors confirm that witnessed verbal consent for submission and publication of this case report including images and associated text has been obtained from the patient. This has been discussed with the editors.
