# Supplementary figures and images for: Unexpected twist: large marginal branch occlusion of the left circumflex artery unveiled by immediate and pivotal cardiac magnetic resonance imaging in a 19-year-old with suspected myocarditis—a case report
Source: Eur Heart J Case Rep. 2026 Jan 22;10(3):ytag015. doi: 10.1093/ehjcr/ytag015 (PMC12962232; doi:10.1093/ehjcr/ytag015)

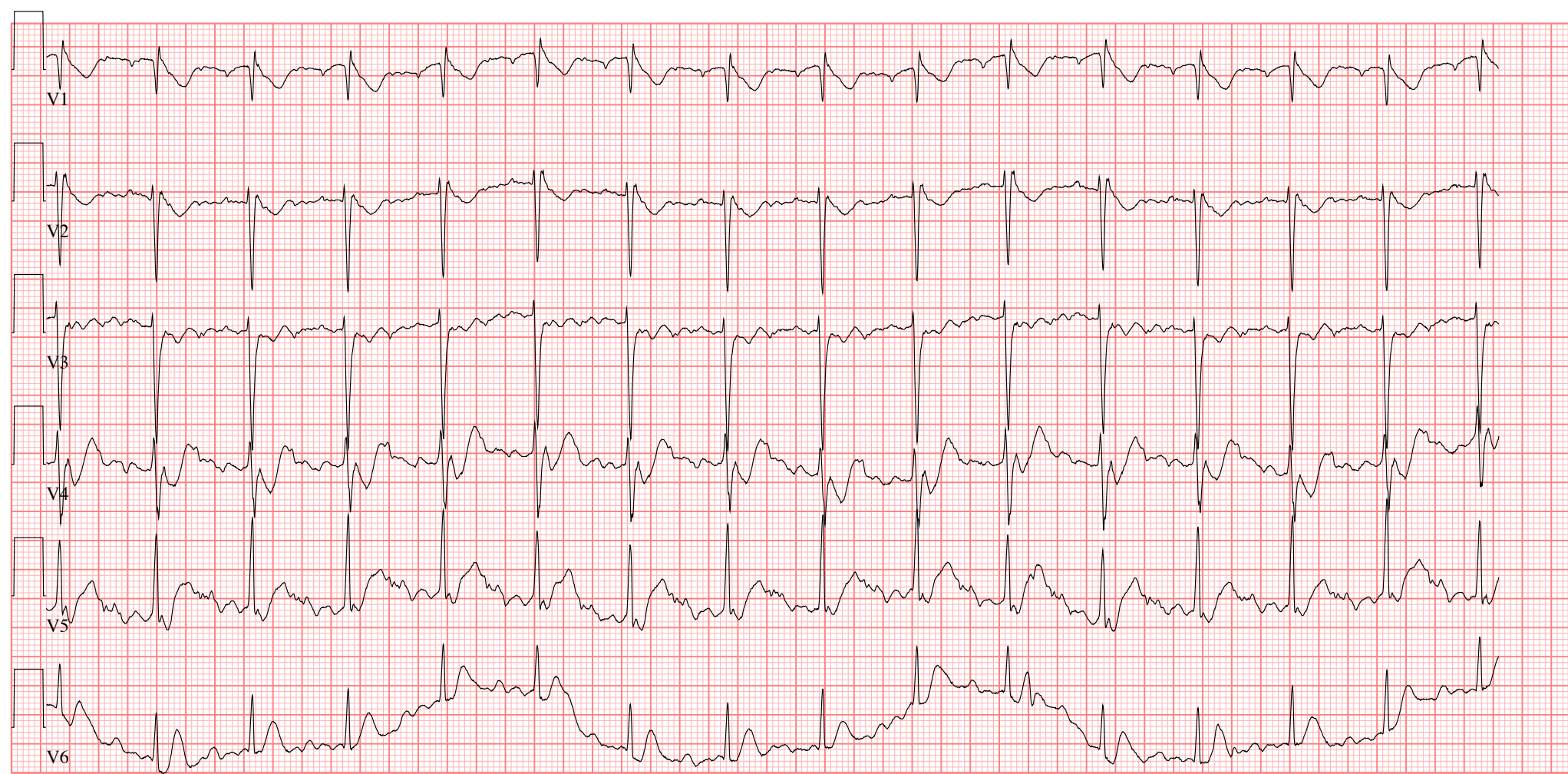

25mm/s 10mm/mV 150Hz

Supplement: ytag015_Supplementary_Data [file ytag015_supplementary_data.zip › ekg_er_precordial.pdf]
