# Supplementary material for: Unexpected twist: large marginal branch occlusion of the left circumflex artery unveiled by immediate and pivotal cardiac magnetic resonance imaging in a 19-year-old with suspected myocarditis—a case report
Source: Eur Heart J Case Rep. 2026 Jan 22;10(3):ytag015. doi: 10.1093/ehjcr/ytag015 (PMC12962232; doi:10.1093/ehjcr/ytag015)
Supplement: ytag015_Supplementary_Data [file ytag015_supplementary_data.zip › EHJ-CR Lack of written consent form V1.0 20200521 2025-05-19.doc]

**Lack of written consent form**

This form should be used by authors wishing to submit a case report/case series/grand round/images in cardiology article to *European Heart Journal – Case Reports* where written consent is not available for a patient included in the manuscript.

The *Editorial Board* of *European Heart Journal – Case Reports* believes that patients should consent to the publication of their cases. However, it is also appreciated that there are some circumstances where the ability to gain informed consent is not possible or appropriate.

Please use the lack of written consent flow chart to confirm the action that is required. This form should only be used as outlined in this flowchart.

**Manuscript Title:** Unexpected Twist: LCX Occlusion Unveiled by Immediate and Pivotal Cardiac MRI in a 19-Year-Old with Suspected Myocarditis

**Manuscript ID (if known):** …………………………………………………………………………………………………………………

**Scenario A – Witnessed verbal consent to publish has been obtained from the patient but written consent is not possible**

*A.1. Please outline the reason(s) why written consent from the patient has not been possible in this situation:*

The patient was first contacted at the end of March and expressed being overwhelmed by his current life circumstances. Although he confirmed having received the written consent documents by post and stated his intention to return them, he did not follow up. After several unsuccessful attempts, I was finally able to reach him again by phone on May 16th. During this very friendly and open conversation, the patient clearly stated that he remembers our previous discussion, is fully aware of the content and purpose of the case report, and gives his verbal consent to publication. He explicitly expressed that he has no objections but still feels unable to deal with paperwork or return the signed forms due to personal stressors.

*A.2 Who witnessed the verbal consent*

Dr. Friederike Noske

A.3 *If possible, please provide the date of the witnessed consent*

16 May 2025

**Scenario B – Verbal consent to publish has been obtained from the patient but not witnessed and written consent is not possible**

*B.1. Please outline the reason(s) why written consent from the patient has not been possible in this situation:*

……………………………………………………………………………………………………………………………………………………………

……………………………………………………………………………………………………………………………………………………………

……………………………………………………………………………………………………………………………………………………………

*B.2. Please outline the reason(s) why witnessed verbal consent from the patient has not been possible in this situation:*

……………………………………………………………………………………………………………………………………………………………

……………………………………………………………………………………………………………………………………………………………

……………………………………………………………………………………………………………………………………………………………

**Scenario C – The patient is deceased and while there are next-of-kin/surviving relatives, it is not possible to contact them.**

*C.1. Please outline the reason(s) why the next-of-kin/surviving relative(s) cannot be contacted in this situation:*

……………………………………………………………………………………………………………………………………………………………

……………………………………………………………………………………………………………………………………………………………

……………………………………………………………………………………………………………………………………………………………

*C.2. If the next-of-kin/surviving relatives contact details are not available, please outline what attempts have been made to obtain these details:*

……………………………………………………………………………………………………………………………………………………………

……………………………………………………………………………………………………………………………………………………………

……………………………………………………………………………………………………………………………………………………………

**Scenario D – The patient is deceased and has no surviving relatives / next-of-kin.**

*D.1. Please provide details of this scenario and how it has been confirmed that there are no appropriate surviving relatives to contact:*

……………………………………………………………………………………………………………………………………………………………

……………………………………………………………………………………………………………………………………………………………

……………………………………………………………………………………………………………………………………………………………

**Scenario E – The patient is alive but has not been contacted to seek consent to publish due to legal or ethical restrictions.**

*E.1. Please provide details of the legal and/or ethical restrictions that prevent contact with the patient to seek consent to publish:*

……………………………………………………………………………………………………………………………………………………………

……………………………………………………………………………………………………………………………………………………………

……………………………………………………………………………………………………………………………………………………………

**Scenario F – The patient is alive but has not been contacted to seek consent to publish and there are no contact details for the patient.**

*F.1. Please provide details of the steps taken by the authors to obtain contact details for the patient:*

……………………………………………………………………………………………………………………………………………………………

……………………………………………………………………………………………………………………………………………………………

……………………………………………………………………………………………………………………………………………………………
